# Adapting Artificial Dentures

**Published:** 1898-07

**Authors:** A. O. Hunt

**Affiliations:** Chicago, Ill.


ARTICLE IV.

### ADAPTING ARTIFICIAL DENTURES.

DR. A. O. HUNT, CHICAGO, ILL.

After a denture has been worn for a long time with only a partial antagonism with a few apposing teeth, the difficulties are multiplied. So also when the mouth is edentulous and no denture has been worn.

When the best model obtainable is at hand, a careful digital examination should first be made of the surface of the mouth, to locate and define the depth and form of the soft spaces within the area to be covered by the plate. These spaces and their form and depth should be outlined on the model, the depth indicated by figures, as it varies greatly in corresponding locations in the same mouth.

The model should then be scraped away in the various places to the proper depth, so that the denture will bear evenly over its whole surface, keeping in mind that the central or palatal portion will not change materially while the alveolar ridge is constantly changed by resorption.

For the upper jaw there are five localities where the denture will find a firm and reasonably permanent rest without excessive pressure on the parts, namely:

The palatal surface, two on the labial surface immediately over the region occupied by the cuspids, and two over the malar processes extending to and above the maxillary tuberosities.

For the lower jaw there are four localities for a firm rest for the plate, two on the labial in the region of the cuspids and two on the posterior buccal margins between the summit of the alveolar ridge and the attachment of the buccinators to the inferior maxillary. The greatest interference in retaining a denture in its place, as they are usually constructed, consists in the action of the muscles on the margins of the plate to push it either up or down, or oftentimes the posterior margin of the upper one extends on the soft palate and is thrown down by its movement; or in the lower denture the posterior ends of the plate are so long that in opening the mouth the stretching of the tissues in front of the ramus move it forward.

While all the muscles may throw a denture out of position, yet by a proper form of the margins, some of them may be utilized as the most important force for retention; notably the buccinators and the orbicularis oris.

A careful examination of the attachments of these muscles should be made, and in shaping the margins their movements should be opposed to each other in such a way that they will be compressed on the denture and hold it firmly.

If improperly made the patient never gets the use of dentures till they learn to hold them in place by the buccinators assisted by the tongue and the orbicularis oris.

With an even pressure over the entire surface of the plate in contact with the mouth, a free movement of the anterior frenum and the anguli oris muscles, the anterior border of the buccinator, the compression of the buccinator at the posterior borders and the compression of the orbicularis oris at the anterior portions, a free movement of the lingual and sub-lingual muscles on the interior surface of the lower denture, with plenty of room for the tongue, accompanied with a stable pressure on the hard or permanent parts of the mouth will produce a perfect adaptation and retention of an artificial denture.—*Dental Review.*

